# Enhanced renoprotective effect of GDNF-modified adipose-derived mesenchymal stem cells on renal interstitial fibrosis

**DOI:** 10.1186/s13287-020-02049-z

**Published:** 2021-01-07

**Authors:** Shulin Li, Yanping Wang, Zhuojun Wang, Lu Chen, Bangjie Zuo, Caixia Liu, Dong Sun

**Affiliations:** 1 Department of Nephrology, Affiliated Hospital of Xuzhou Medical University, 99 West Huai-hai Road, Xuzhou, 221002 Jiangsu China; 2 Department of Internal Medicine and Diagnostics, Xuzhou Medical University, Xuzhou, 221002 China

**Keywords:** Mesenchymal stem cells, Glial cell line-derived neurotrophic factor, Renal interstitial fibrosis, Microvascular injuries, PI3K/Akt/eNOS

## Abstract

**Background:**

The therapeutic effect of mesenchymal stem cells (MSCs) from human adipose tissue on renal interstitial fibrosis has been demonstrated by several groups. However, the way to enhance the renoprotective effect of adipose-derived mesenchymal stem cells (AMSCs) and the possible mechanisms are still unclear. The present study aimed to determine whether glial cell line-derived neurotrophic factor (GDNF)-modified AMSCs hold an enhanced protective effect on renal fibrosis.

**Methods:**

AMSCs were isolated and purified for culture. The gene GDNF has been constructed to transfect into AMSCs. The ability of GFP-AMSCs and GDNF-AMSCs supernatants to promote tube formation of endothelial cells, repair damaged endothelial cell junctions, and improve endothelial cell function was compared by using tube formation assay, immunofluorescence techniques, and vascular ring assay, respectively. Furthermore, HE and Masson staining were used to observe the histological morphology of the kidney in vivo. Peritubular capillary changes were detected and analyzed by fluorescence microangiography (FMA). Meanwhile, the hypoxia, oxidative stress, fibrotic markers, and PI3K/Akt pathway proteins were measured by western blot or qRT-PCR technics.

**Results:**

Compared with GFP-AMSCs only, GDNF-AMSCs could enhance the repair of injured endothelial cells and promote angiogenesis through secreting more growth factors in the supernatant of GDNF-AMSC culture media demonstrated in vitro studies. Studies in vivo, unilateral ureteral obstruction (UUO)-induced mice were injected with transfected AMSCs through their tail veins. We showed that enhanced homing of AMSCs was observed in the GDNF-AMSC group compared with the GFP-AMSC group. The animals treated with GDNF-AMSCs exhibited an improvement of capillary rarefaction and fibrosis induced by obstructed kidney compared with the GFP-AMSC group. Furthermore, we reported that GDNF-AMSCs protect renal tissues against microvascular injuries via activation of the PI3K/Akt signaling pathway. Therefore, GDNF-AMSCs further ameliorated the tissue hypoxia, suppressed oxidative stress, and finally inhibited endothelial to mesenchymal transition noting by decreased coexpression of endothelial cell (CD31) and myofibroblast (a-SMA) markers.

**Conclusion:**

Collectively, our data indicated that the GDNF gene enhances the ability of AMSCs in improving renal microcirculation through PI3K/Akt/eNOS signaling pathway and afterward inhibit the EndMT process and kidney fibrogenesis, which should have a vast of implications in designing future remedies for chronic kidney disease (CKD) treatment.

## Background

The number of CKD patients and those progressing to end-stage renal disease (ESRD) has been increased in recent years [1]. The characteristics of renal fibrosis are hypoxia, oxidative stress, inflammatory cell infiltration, fibroblast activation, and extracellular matrix accumulation. These mentioned characteristics are the common pathogenic pathway of progressive CKD renal disease [2–4]. Although many reasons are inducing renal fibrosis, capillary rarefaction secondary to endothelial injury represents a major cause contributing to tissue damage and loss of renal function [5, 6]. The kidney is a rich vascular structure and a highly vascularized viscus. Therefore, it is essential to maintain the blood supply for the kidneys and tissue. The loss of capillaries results in the imbalance of local tissue oxygen demand and supply, which causes renal fibrosis and further leads to a series of events, such as the accumulation of reactive oxygen metabolites and EndMT.

Stem cell-based therapy is not only the most advanced regenerative therapy but one that has shown great promise for the treatment of CKD [7]. Derivation of mesenchymal stem cells (MSCs) from human adipose avoids several ethical concerns of embryonic stem cells and teratogenicity potential of induced pluripotent stem cells [8–10]. The therapeutic effects of AMSCs on kidneys could be mediated not only through the direct differentiation of AMSCs into target cells but also through the autocrine- or paracrine-mediated release of angiogenic cytokines and growth factors, which then improve endogenous repair and mitigate the fibrotic kidneys.

Glial cell line-derived neurotrophic factor (GDNF) is regarded as one of the most potent neurotrophic factors for neuronal diseases for a long time [11–14]. A report confirmed neuroprotective effects on hypoxic-ischemic encephalopathy (HIE) of GDNF through activating RET/PI3K/Akt signaling pathway [15]. In a recent study, another research demonstrated that GDNF−/− mutant mice showed kidney agenesis and dysgenesis, suggesting that GDNF plays a critical role in the development of renal system [16]. According to our previous studies [17–19], we found that GDNF is a tissue morphogen affecting stem cells, which could enhance migration and differentiation of stem cells and finally reverse the kidney injury. Therefore, we speculate that pretreatment with the GDNF gene on AMSCs could better alleviate renal fibrosis by strengthening the ability of AMSCs differentiating into endothelial cells, promoting AMSCs homing to damaged kidney, enhancing angiogenesis through PI3K/AKT/eNOS pathway, and further suppressing oxidative effect and EndMT.

## Materials and methods

### Ethical statement

Study methods were performed according to the relevant guidelines and regulations. Human AMSC samples were collected with the written consent of all subjects, and the protocol was approved by the Institutional Review Board of the Affiliated Hospital of Xuzhou Medical University. All experiments involving animals followed the animal use protocol enacted by the Institutional Animal Care and Use Committee of Xuzhou Medical University (permit number: SYXK2015-0030).

### Cell culture

The cells were obtained from human abdominal subcutaneous adipose tissues and cultured in a modified minimum essential medium (Hyclone, Logan, NY, USA) supplemented with 10% fetal bovine serum (FBS; Hyclone, Logan, UT, USA) and 1% Mycillin (Beyotime, Shanghai, China), plated in 25 cm^2^ T-flasks, and maintained at 37 °C in a 5% CO2 incubator. The culture medium was changed every 2 or 3 days.

### Lentivirus vector production

A green fluorescent protein (GFP) label for a lentivirus vector plasmid system carrying the GDNF gene was constructed by the Ji Kai Gene Company (Shanghai, China). AMSCs at the third passage were transfected with adenovirus vectors at the most appropriate multiplicity of infection (MOI) = 20 following standard procedures. GFP expression was observed and detected at 1 day, 2 days, and 3 days after lentiviral vector transfection.

### Flow cytometry

For MSCs’ characterization, cells were analyzed by flow cytometry (BD, Franklin Lakes, NJ, USA). Cells were detached by treatment with 0.25% trypsin/EDTA, washed with (PBS), and incubated in the dark for (30 min) at room temperature with the hematopoietic and mesenchymal antibodies, PE-CD34, FITC-CD45, FITC-HLA-DR, APC-CD105, PE-Cy7-CD90, and PE-CD73 (BD, USA). Approximately 1 × 10^4^ cells were then washed with wash flow buffer and re-suspended in (500 μl of 1%) formaldehyde solution, examined using flow cytometer, and analyzed by Flowjo software (Flowjo, Ashland, Oregon, USA).

### Tube formation assay

Tube formation test was performed as previously described [20]. In brief, endothelial cells (1 × 10^4^) were seeded in a 48-well plate coated with 100 μl of growth factor-reduced Matrigel TM (BD, USA) and incubated with basic culture medium, GFP-AMSC culture medium, and GDNF-AMSC culture medium for tube stabilization for 24 h at 37 °C to allow for gelling. Tube formation was observed and quantified by measuring the total tube loops in five random microscopic fields with a computer-assisted microscope (Olympus, Japan).

### Vascular ring assay

We removed the thoracic aorta after euthanasia. The vessels were dissected free of fat and connective tissue and cut into ring segments approximately 2–3 mm in width. The dissection was performed on the tension transducer in the groove full of Krebs solution (K3753, Sigma) and was maintained at 37 °C. The aortic segments pretreated with AMSC culture medium or GDNF-AMSC culture medium were equilibrated for 90 min and following which they were exposed to standard concentrations of KCl (60 Mm) for two times. KCl is washed away immediately after the vasoconstriction is stable. The aortic segments were equilibrated for 20 min; afterward, the vasoconstriction was stimulated by phenylephrine (PE) (10^−6^ mol/L). After the vasoconstriction was stabilized, the vasodilatation was administered sequentially with acetylcholine (Ach) at a cumulative concentration of 10^−8^ mol/L–10^−4^ mol/L and was recorded.

Vasodilatation rate = (the maximum tension before Ach administration − the tension recorded after Ach administration)/(the maximum tension before Ach administration − the basic tension) × 100%

### Endothelial junctions test

GFP-AMSC culture medium and GDNF-AMSC culture medium were generated as follows: GFP-AMSCs and GDNF-AMSCs were cultured in culture flasks. Afterward, culture media from passages 3–5 were harvested and then stored at − 80 °C until use. HUVECs were cultured in 4 conditions, normoxia condition, hypoxia condition for 12 h, preconditioned with the GFP-AMSC culture media, and GDNF-AMSC culture media under hypoxic condition. Then, the immunofluorescence staining was performed. The procedure is the same as that described in FMA.

### In vivo tracking of AMSCs

Green fluorescence protein (GFP)-labeled AMSCs (5 × 10^5^) were intravenously injected after the UUO model established. The kidney samples were harvested at days 3 and 7 and embedded by optimum cutting temperature (OCT) after fixation and dehydration. OCT-embedded kidneys were sectioned into 10 μm sections and mounted on Superfrost slides to follow the localization of MSCs in kidneys. Slides were stained with DAPI (4′6-diamidino-2-phenylindole) and were observed under a confocal laser microscope (FV1000; Olympus, Tokyo, Japan).

### Animal models

Nu/nu mice (body weight 18–22 g, age 6–8 weeks) were purchased from the Laboratory Animal Centre of Xuzhou Medical University (Jiangsu, China). The animals were maintained at a temperature (22 ± 1 °C) and lighting (12 h light-dark cycle) controlled room with free access to food and water. After 1 week of acclimatization, the mice were randomly divided into four following groups (*n* = 10 in each group): sham-operated mice (sham group), UUO mice treated with intravenous injection of saline solution (UUO group), UUO mice treated with intravenous injection of GFP-AMSCs immediately after model establishment (GFP-AMSC group), and UUO mice treated with intravenous injection of GDNF-modified AMSCs immediately after the model was established (GDNF-AMSC group). UUO was performed using an established procedure as described [21]. Mice were anesthetized with chloral hydrate (10.0%, 0.003 ml/g intraperitoneal) and sacrificed by sodium pentobarbital injection. The mice in the GFP-AMSC group were intravenously injected through the tail vein with 5 × 10^5^ GFP-labeled AMSCs in 150 μl of saline solution, and the GDNF-AMSC group was injected with 5 × 10^5^ GFP-labeled GDNF-AMSCs in 150 μl of a saline solution via the same route [22, 23]. As a control, we injected the mice in the UUO group with a saline solution.

On days 3 and 7 post-surgery, we sacrificed mice from four groups. Mice blood was collected from the retro-orbital plexus to measure the renal function, including serum creatinine (Scr) and blood urea nitrogen (BUN). Then, the left kidneys were respectively extracted and decapsulated in saline solution. One part of the kidneys was fixed in 10% formaldehyde for the pathological test, while others stored at − 80 °C for later PCR and Western blot analysis.

### Renal histology

For evaluating renal morphology, we prepared paraffin-embedded mouse kidney sections (4-μm-thick sections) by a routine procedure. We stained these sections with hematoxylin-eosin and Masson’s trichrome by standard protocol. The areas of interstitial fibrosis were detected using Masson’s trichrome staining, which was stained dark blue. Ten microscopic visual fields of renal tissues were selected randomly in the sections under high-power magnification (× 40).

### FMA

FMA was performed as previously described [24]. Briefly, mice were anesthetized with chloral hydrate (10.0%, 0.003 ml/g intraperitoneal) in a supine position. The thoracic cavity was exposed after cutting along bilateral ribs. All solutions were prewarmed to 41 °C according to Rafael Karmann et al. One milliliter of heparinized saline followed by 1 ml of 3 M KCl was injected in the beating left ventricle using a vein needle. The right atrium was then cut and the mouse was perfused with 41 °C prewarmed PBS (10 ml), immediately followed by 5 ml of the agarose-microbead mixture (500 ml 0.02 mm FluoSpheres plus 4.5 ml 1% agarose/mouse). The kidneys were removed after perfusion for about 1 min and carefully placed in a small container surrounded by ice for 10 min. Thereafter, the kidneys were fixed in 4% paraformaldehyde on ice for 2 h, and then incubated in 30% sucrose in PBS at 4 °C overnight and embedded by OCT. OCT-embedded kidneys were sectioned into 10 μm sections and mounted on Superfrost slides. For immunofluorescence staining, selected sections were blocked with 10% donkey serum containing 0.4% Triton-X-100 for 1 h, and then the slides were respectively immunostained with primary antibodies, including anti-CD31 rabbit antibody (1:20, ab28364, Abcam), anti-alpha smooth muscle actin rabbit antibody (1:100, ab32575, Abcam), anti-alpha smooth muscle actin mouse antibody (1:200, ab7817, Abcam), Anti-VE-Cadherin antibody (1:200, ab33168, Abcam). To visualize the primary antibodies, slides were stained with Donkey anti-Mouse IgG (H+L) Highly Cross-Adsorbed Secondary Antibody, Alexa Fluor 488 (1:2000, #A-21202, ThermoFisher), and Goat anti-Rabbit IgG (H+L) Highly Cross-Adsorbed Secondary Antibody, Alexa Fluor 594 (1:200, #A-11037, ThermoFisher). Thereafter, all slides were viewed with a confocal laser microscope (FV1000; Olympus, Tokyo, Japan).

### Western blot

Kidney tissue samples were lysed in RIPA and PMSF on ice for 30 min and then centrifuged at 12,000 rpm for 15 min at 4 °C. After the protein samples were heated in boiling water for 5 min, approximately 50 μg of total protein was loaded on 8%, 10%, or 12% sodium dodecyl sulfate-polyacrylamide (SDS) gels and transferred to a polyvinylidene difluoride (PVDF) membrane by electroblotting. Non-specific binding was blocked by incubating the membrane in 5% non-fat dry milk for 1 h at room temperature. The membrane was then incubated with primary antibodies against HIF-1α (1:500, 20960-1-AP, Proteintech), gp91-phox (1:800, ab180642, Abcam), p67-phox (1:2000, sc-374510, santa cruze), VEGF1(1:200, ab39256, Abcam), TGF-β1(1:500, ab92486, Abcam), α-SMA (1:1000, #19245, Cell Signaling Technology), p-enos (1:1000, #9570, Cell Signaling Technology), enos (1:1000, #32027, Cell Signaling Technology), PI3K (1:1000, #4249,Cell Signaling Technology), p-Akt (1:2000, #4060, Cell Signaling Technology), and Akt (1:1000, #9272, Cell Signaling Technology) at 4 °C overnight, followed by an incubation with ECL secondary antibodies. The signal was detected with ImageQuant LAS 4000 mini and was quantified by beta-actin.

### Quantitative RT-PCR

RNA from kidney samples was isolated using the RNeasy kit (Roche, USA). Quantitative RT-PCR was carried out using an ABI PRISM 7300 Sequence Detection System. The final reaction contained template complementary DNA, iTaq SYBR Green Supermix with ROX (Bio-Rad, Hercules, CA, USA) and gene-specific primers. The sequences of the mouse primer pairs used in qRT-PCR are as follows: TGF-β1 (mouse) forward primer, 5′-TCGCTTTGTACAACAGCACC-3′, reverse primer, 5′-ACTGCTTCCCGAATGTCTGA-3′; HIF-1 a (mouse), forward primer, 5′-CTCACCAGACAGAGCAGGAA-3′, reverse primer, 5′-AAGGGAGCCATCATGTTCCA-3′; gp91-phox (mouse), forward primer, 5′-GAGGTTGGTTCGGTTTTGGC-3′, reverse primer, 5′-TGCACAGCAAAGTGATTGGC-3′; p67-phox (mouse), forward primer, 5′-AACATAGGCTGCGTGAACACTATCC-3′, reverse primer, 5′-GCAAGGTCGTACTTCTCCATTCTGTAG-3′. The conditions 50 °C for 2 min and 95 °C for 10 min followed by 40 cycles for 30 s at 95 °C, 45 s at 60 °C, and 30s at 72 °C were applied. GADPH was used as an internal control. The cycle threshold values of GADPH and other specific genes were acquired after PCR. The normalized fold expression was obtained using the 2−ΔΔCT method. The results were expressed as the normalized fold expression for each gene.

### Statistical analyses

Data were presented as the mean ± SD, and statistical analysis was performed with GraphPad Prism software, version 6.0c. Comparisons between groups were analyzed by one-way analysis of variance (ANOVA) with Turkey’s or Dunnett’s post hoc test or post hoc Bonferroni correction. A *P* value less than 0.05 was considered statistically significant.

## Results

### Cell culture and cell characterization of human AMSCs

The cells were obtained from human abdominal subcutaneous adipose tissues and cultured in a modified minimum essential medium (Hyclone, Logan, NY, USA) supplemented with 10% fetal bovine serum (FBS; Hyclone, Logan, UT, USA) and 1% Mycillin (Beyotime, Shanghai, China), plated in 25cm^2^ T-flasks, and maintained at 37 °C in a 5% CO2 incubator.

**Fig. 1:**
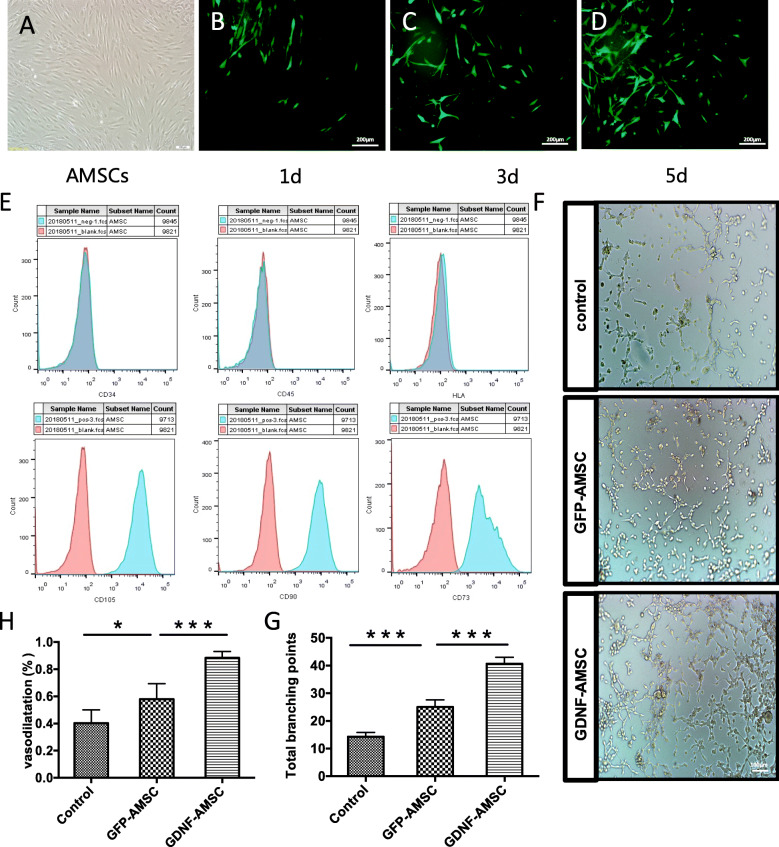
Cell characterization of AMSCs and angiogenic capacity of GDNF-AMSCs in vitro. **a** Morphology of AMSCs after 3 passages under high magnification. **b**–**d** Fluorescence expression of lentiviral vectors with GDNF-transfected AMSCs at 1 day, 3 days, and 5 days. Low GFP expression, indicated by green fluorescence, was detected at 1 day and gradually increased over the course of the transfection. **e** The cell surface markers of AMSCs were identified by flow cytometry. CD105, CD90, and CD73 were highly expressed in the third generation of AMSC. The expressions of CD34, CD45, and HLA-DR were negative. **f**, **g** The tube-like structures were observed using a microscope after 12 h. Representative images and quantification of the organization into capillary-like structures in each group. **h** Bar graph showing the vasodilation effects after AMSCs and GDNF-transfected AMSC treatments (*P < 0.05, **P < 0.01, ***P < 0.001)

The expressions of cell surface markers on the isolated AMSCs were evaluated by flow cytometry (Fig. 1e), showing representative flow cytometry analysis for AMSCs. The cells were positive for the MSC markers CD105, CD90, and CD73 but were negative for the hematopoietic marker CD34, CD45, and HLA-DR, demonstrating that the extracted cells are MSCs.

### Lentivirus vector production

A green fluorescent protein (GFP) label for a lentivirus vector plasmid system carrying the GDNF gene was constructed by the Ji Kai Gene Company (Shanghai, China). AMSCs at the third passage were transfected with adenovirus vectors at the most appropriate multiplicity of infection (MOI) = 20 following standard procedures. To determine the transfection efficiency, we used fluorescence microscopy to observe the green fluorescent protein (GFP) signal. A faint GFP signal was detected on day 1, and it gradually increased over the course of the transfection. By day 5 after transfection, approximately 80% of AMSCs expressed GFP, as indicated by green fluorescence (Fig. 1b–d).

### GDNF-AMSCs promote capillary-like formation in vitro

A Matrigel-based tube formation assay was performed to assess the capacity of HUVEC for endothelial angiogenesis. In the basic culture medium, rarely formed capillary-like structures were observed. The total number of tubes in the tubular network remarkably increased upon GFP-AMSCs-culture media (GFP-AMSCs-CM) treatment compared with the control group (*P* = 0.0025), and GDNF-AMSC-CM application significantly improved tube formation capacity compared with the GFP-AMSC group (*P* = 0.0003). These results indicated that GDNF-AMSCs play an important role in angiogenic activity (Fig. 1f, g).

### GDNF-AMSCs improve vasodilation ability of blood vessels

The exposure of the isolated thoracic aorta to (PE) resulted in a concentration-dependent contraction, and the maximum tension was recorded. Vasodilative tension was recorded after administered sequentially with acetylcholine (Ach) at a cumulative concentration of 10^−8^ mol/L–10^−4^ mol/L. Vasodilatation rate was calculated according to the formula mentioned in the method. As shown in Fig. 1h, GFP-AMSCs could increase the vasodilation ability of blood vessels compared with the control group (*P* = 0.0452). However, exposure to GDNF-AMSC culture media resulted in more robust vasodilatation compared with the GFP-AMSC treatment (*P* = 0.0004), which may increase blood flow and finally improve the renal microcirculation.

### Endothelial differentiation medium affects cell shape

**Fig. 2:**
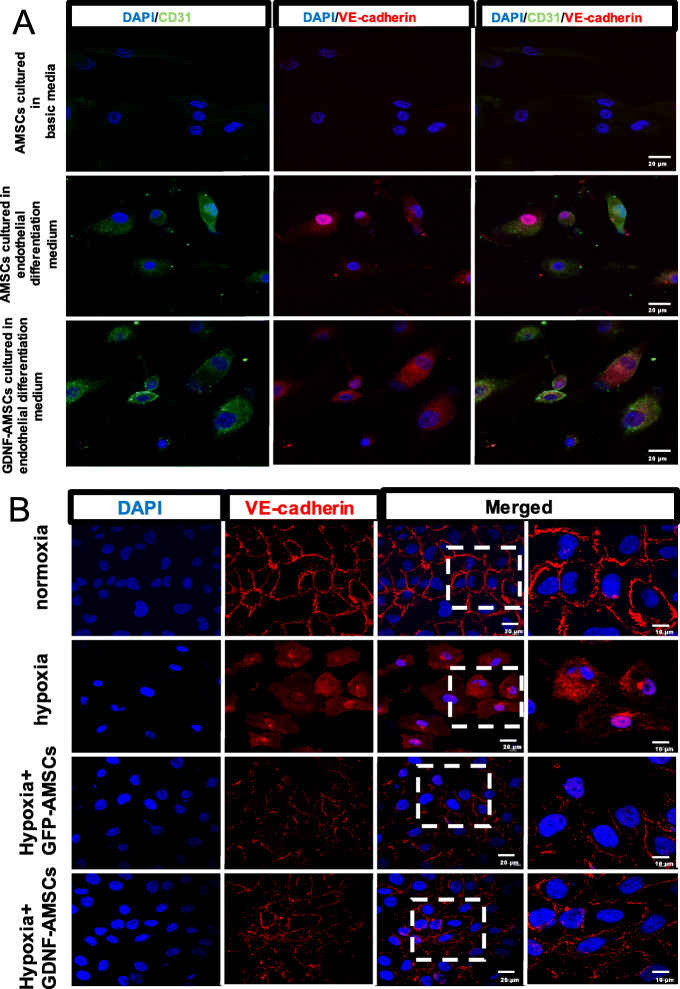
Preconditioning AMSCs with the GDNF gene enhance their differentiation and cell repairment capacity. **a** After the induction by VEGF and FGF for 21 days, morphological changes of cells were observed and fluorescence expressions of endothelial markers (CD31, green and VE-cadherin, red) were detected. Both AMSCs and GDNF-AMSCs can express endothelial cell markers (CD31 and VE-cadherin) after inducted by the endothelial inducer. **b** The expression and the distribution of VE-cadherin (red) were regulated and highly expressed in the normoxia group but was reduced and rearranged in the hypoxia group. The morphology of endothelial cells and cell-cell contact was slightly recovered in the AMSC group and significantly promoted after GDNF-AMSC treatment. Nuclei were stained with DAPI (blue)

Because MSCs display a multiple differentiation capacity in vitro, we decided to explore if AMSCs could differentiate towards an endothelial-like phenotype. After the induction by VEGF and FGF for 21 days, morphological changes of cells were observed and expressions of endothelial markers (CD31 and VE-cadherin) were detected (Fig. 2a). AMSCs cultured in the basic medium were used as the negative control for the expression of endothelial markers. Green and red fluorescences are almost invisible, and the very low fluorescent expressions may be due to non-specific staining or affected by some cytokines secreted by stem cells. However, immunofluorescence staining showed that the observed change of shape of differentiated AMSCs was followed by distinct expressions of endothelial markers after AMSCs cultured in VEGF and FGF medium. The shape of differentiated AMSCs cultured in VEGF and FGF medium showed a cobblestone-like morphology typical for endothelial cells in contrast to fibroblast-like cells cultured in basic medium. The GDNF-AMSC group showed no changes compared with the AMSC group. Collectively, these results confirm the endothelial differentiation of AMSCs and prove that both AMSCs and GDNF modified AMSCs have the potential to repair the microvascular injury.

### GDNF-AMSCs regulate endothelial junctions in vitro

Vascular endothelial cadherin (VE-cadherin) is considered to be a specific representative for endothelial adherens assembly and barrier architecture. HUVECs expressed VE-cadherin consistently in the normoxia group, showing regular and tight cell-cell contacts. However, the expression of VE-cadherin followed a sawtooth distribution typically associated with the lack of tight junctions in the hypoxia group. The strongly expressed VE-cadherin protein throughout the endothelium was dispersed in the cytoplasm and nucleus after 12 h hypoxic condition whereas partially reverse to distribute on the endothelial cell membranes in the GFP-AMSC group. Moreover, endothelial cells’ precondition with the GDNF-AMSC culture media showed a more abundant cell-cell contact, and the morphology of endothelial cells tends to be normal in comparison with those cultured under hypoxic conditions (Fig. 2b). These data indicate that GDNF-AMSCs may help endothelial cells rebuilt the healthy endothelium barrier that can prevent vascular permeability.

### Distribution of intravenously injected AMSCs

**Fig. 3:**
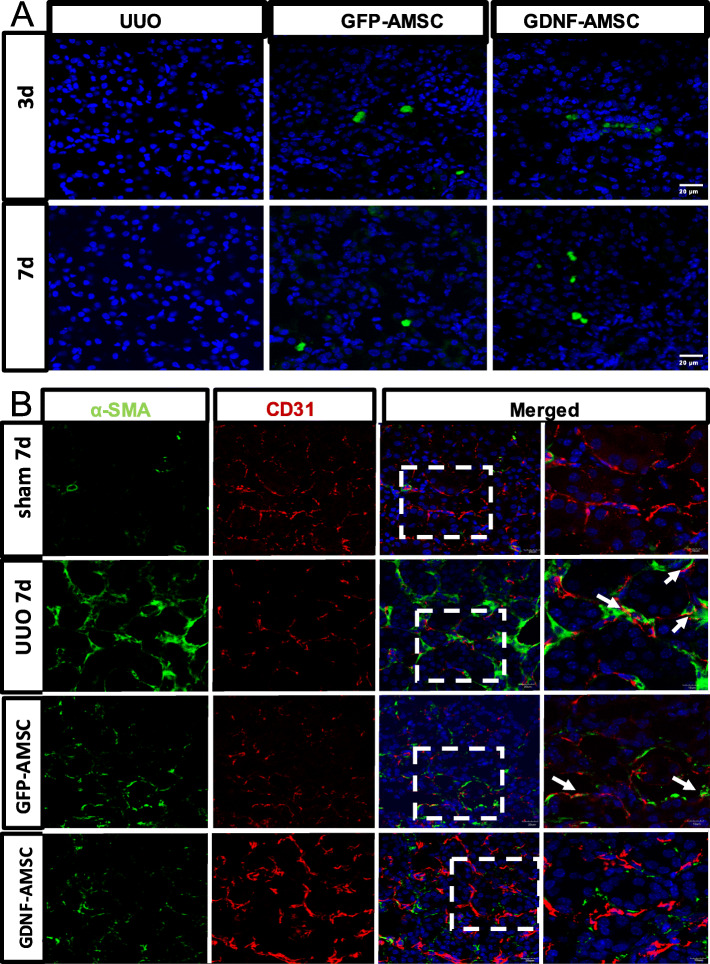
The distribution of GDNF-AMSCs in the injured kidney and GDNF-AMSCs inhibits EndMT progression in the obstructed kidney. **a** Intravenously delivered GFP-labeled AMSCs (green) were detected in the obstructed kidneys at days 3 and 7, and GFP-labeled GDNF-AMSCs (green) were more detected by laser confocal microscopy. Nuclei were stained with DAPI (blue). **b** There was no evident two-color immunofluorescence that identifies EndMT cells that coexpress endothelial cells (CD31, red) and myofibroblast cells (a-SMA, green) shown in the sham group. EndMT progression was increased in the UUO group and decreased in the AMSC and GDNF-AMSC group, especially in the GDNF-AMSC group, indicating by the coexpression of CD31+a-SMA+. White arrows indicate positive coexpressing staining. Nuclei were stained with DAPI in blue. Bar = 20 μm

To explore the effect and mechanism of stem cells in vivo, GFP-AMSCs and GDNF-AMSCs were respectively injected into the classic mice model of renal fibrosis. The distribution of AMSCs was closely related to their functional mechanism. To evaluate the transplantation efficiency of GFP-labeled AMSCs in the UUO model, stem cells were detected in frozen samples by a laser confocal microscope. As shown in Fig. 3a, the tissue showed no signal of GFP in the UUO group at days 3 and 7. However, signals were successfully detected in the GFP-AMSC group, which suggested that AMSCs injected via the tail vein could enter to the injured kidney through the blood circulation. Moreover, stronger signals were observed in the GDNF-AMSC group, indicating that GDNF could enhance AMSCs homing to the injured kidney.

### GDNF-AMSCs alleviate renal interstitial fibrosis

**Fig. 4:**
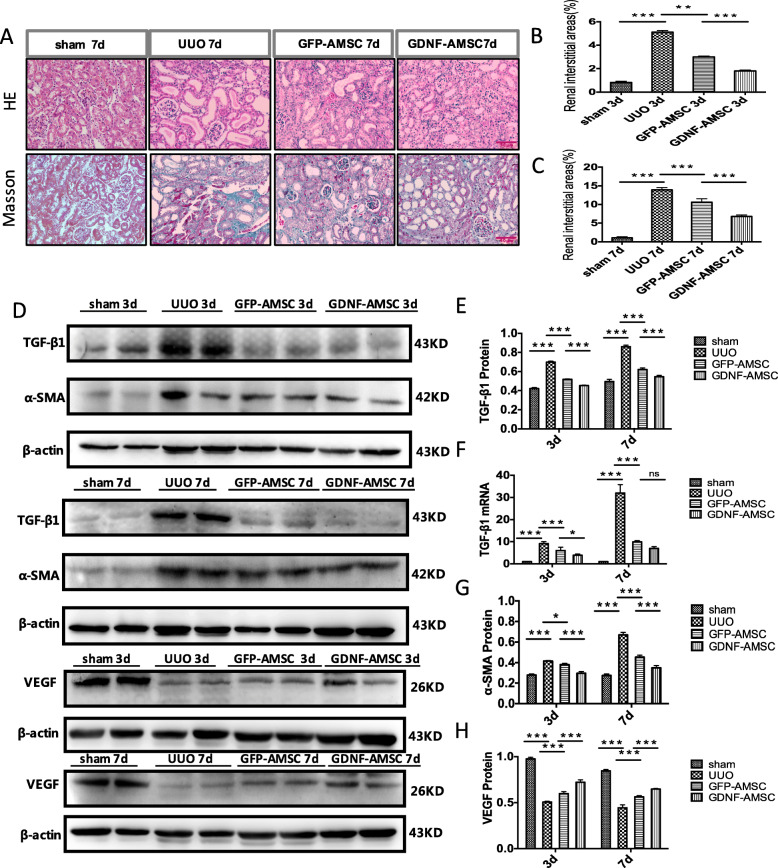
Renal histological changes and gene and protein expressions of fibrotic and angiogenic markers. **a** Representative micrographs of hematoxylin-eosin and Masson’s trichrome demonstrate histological changes in the 7 days post-surgery groups. Bar = 20 μm. **b**, **c** Quantification of renal interstitium areas in each group on both 3 and 7 days post-surgery (*P < 0.05, **P < 0.01, ***P < 0.001). **d**–**h** TGF-β1, a-SMA, and VEGF expression were confirmed after GDNF-AMSC treatment. Representative western blot (**d**) and quantitative data for TGF-β1 (**e**), a-SMA (**g**), and VEGF (**h**) are presented, and TGF-β1 mRNA levels were shown by real-time PCR (**f**) (*P < 0.05, **P < 0.01, ***P < 0.001)

Renal histology was investigated using hematoxylin-eosin and Masson’s trichrome staining. There was no significant histological abnormality in the sham group. After unilateral ureteral occlusion, severe morphological lesions and extracellular matrix (ECM) production were detected. Compared with UUO, the GFP-AMSC and GDNF-AMSC groups, especially the latter, showed less renal fibrosis (Fig. 4a–c).

The upregulation of the transforming growth factor-β (TGF-β) gene and protein expressions were detected in the obstructed kidney (*P* < 0.001 vs. sham), demonstrating the progression of renal fibrosis. Mice treated with GFP-AMSCs showed a significant reduction in the degree of fibrosis compared with the UUO group at days 3 and 7 (*P* < 0.001 vs. UUO), while the TGF-β1 expressions were further reduced in the GDNF-AMSC group compared with the GFP-AMSC group (*P* < 0.001 vs. GFP-AMSC) (Fig. 4d–f).

**Fig. 5:**
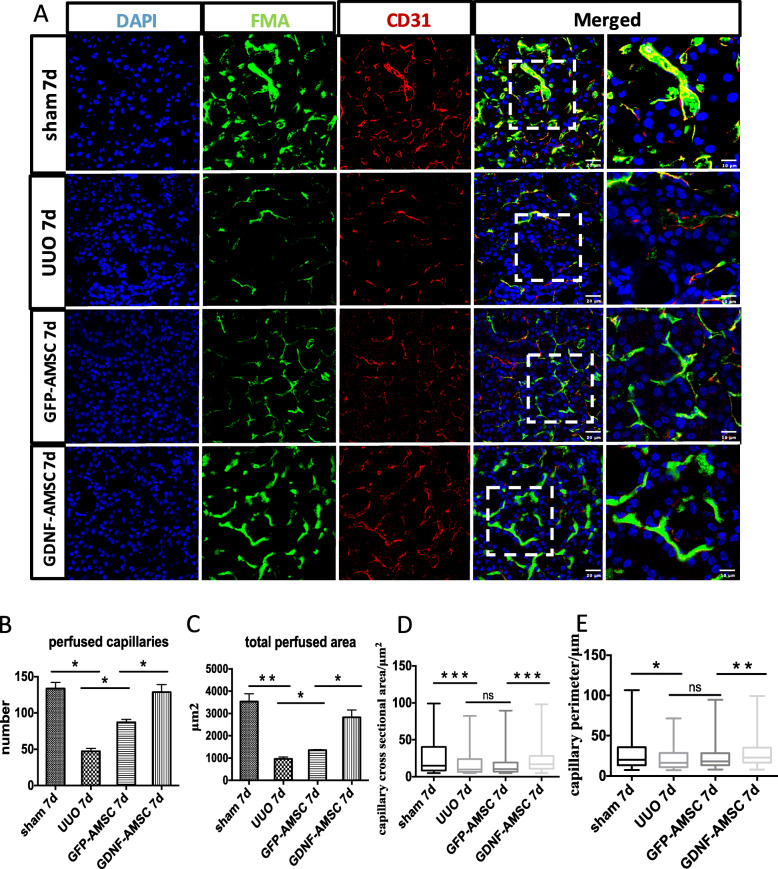
Immunofluorescence staining of α-SMA in the renal mesenchyme and quantification revealed the induction of interstitial fibrosis after unilateral ureteral obstruction compared with the sham group. However, GDNF-AMSCs suppressed fibrotic expressions and increased PTCs density (*P < 0.05, **P < 0.01, ***P < 0.001)

Immunofluorescence staining of alpha-smooth muscle actin (α-SMA) protein expression showed a significant increase in the UUO group accompanied by a decrease of peritubular capillary (PTC) density compared with the sham group, whereas mice treated with GFP-AMSCs or GDNF-AMSCs showed a progressive decrease with significantly less interstitial a-SMA staining at day 3 and 7 post-UUO (Fig. 5). The expression levels of a-SMA in whole kidney extracts were also detected by Western blotting (Fig. 4d, g) and confirmed reduced a-SMA levels in the GDNF-AMSC group (*P* < 0.001 vs. GFP-AMSC). These results provide more direct and precise evidence that GDNF could enhance the ability of AMSCs to repair the renal microvascular injury and subsequently alleviate renal interstitial fibrosis.

### GDNF-AMSCs promote microvascular repair and angiogenesis

Vascular endothelial growth factor (VEGF), a specific endothelial growth factor, was highly expressed in the sham group whereas ureteral ligated mice showed a sharper decline of VEGF expression (*P* < 0.001 vs. sham). However, treating with GFP-AMSCs after 3 or 7 days, VEGF expression was higher compared with that in the UUO group (*P* < 0.001 vs. UUO), and this increase was further augmented in the GDNF-AMSC group at each time point (*P* < 0.001 vs. GFP-AMSCs) (Fig. 4d, h), confirming the better repairment of microvessels of GDNF-modified AMSCs in the renal interstitial fibrosis.

### Peritubular capillary changes detected by fluorescence microangiography (FMA)

**Fig. 6:**
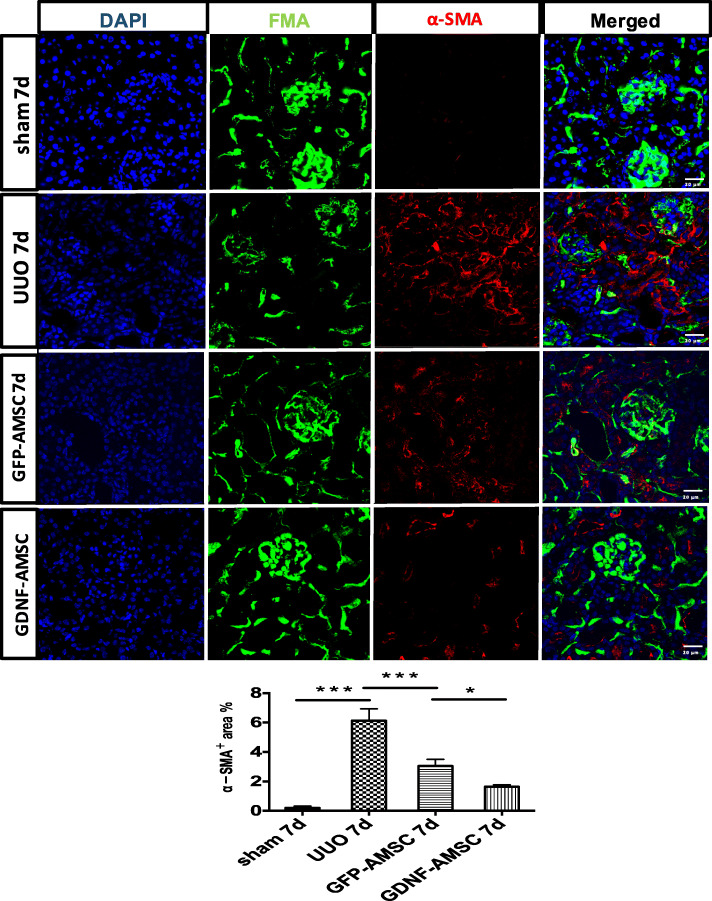
Fluorescence microangiography (FMA). **a**–**c** The green FMA solution injected after sham surgery and UUO 7 days, surrounded with CD31 immunostaining (expressed on the surface of endothelial cells), demonstrates capillary rarefaction in response to the obstructed kidney. The reduced capillary number and perfused area were restored after AMSCs and GDNF-AMSC treatment, especially in the GDNF-AMSC group. Representative images in the box were presented by higher magnification, demonstrating that capillaries surrounded by CD31+ endothelial cells in the UUO group do not appear a luminal FMA signal indicating that these capillaries lack perfusion, but increased FMA perfused area was shown in the GDNF-AMSC group. **d**–**e** Both of the individual capillary cross-sectional area and perimeter in the UUO group were smaller than those in the sham group, but AMSC treatment has no good effect on them. These individual capillary markers were significantly improved after GDNF-AMSC treatment in comparison to the AMSC group (*P < 0.05, **P < 0.01, ***P < 0.001)

To evaluate the renal microvasculature, we applied the FMA method combined with the MATLAB analysis of the peritubular capillary size and density (Fig. 6a–e). The capillary number and total perfused area were reduced in the UUO group compared with the sham group but increased after GFP-AMSC treatment and further increased within the cortical tubulointerstitium in the GDNF-AMSC group, indicating GDNF-AMSCs has a better effect on improving renal microcirculation. Both of the individual capillary cross-sectional area and perimeter in the UUO group were smaller than those in the sham group. However, these individual capillary markers did not significantly change after GFP-AMSC treatment in comparison to the UUO group but were increased significantly in the GDNF-AMSC group, indicating that the single peritubular capillary size was improved after GDNF-AMSC treatment. To mark the outline of microvessels, CD31, an endothelial cell antigen expressed on the endothelial cell surface, was utilized. The magnified images in the right panel showed that capillaries surrounded by CD31+ endothelial cells in the UUO group appeared a decreased luminal FMA signal, suggesting a lack of perfusion of these vessels, but increased FMA perfused area was shown in the GFP-AMSC group, and further increased in the GDNF-AMSC group. All microvascular characteristics were analyzed by MATLAB-based script (Supplementary Fig. S1).

### GDNF-AMSCs activate the PI3K/Akt/eNOS signaling pathway

**Fig. 7:**
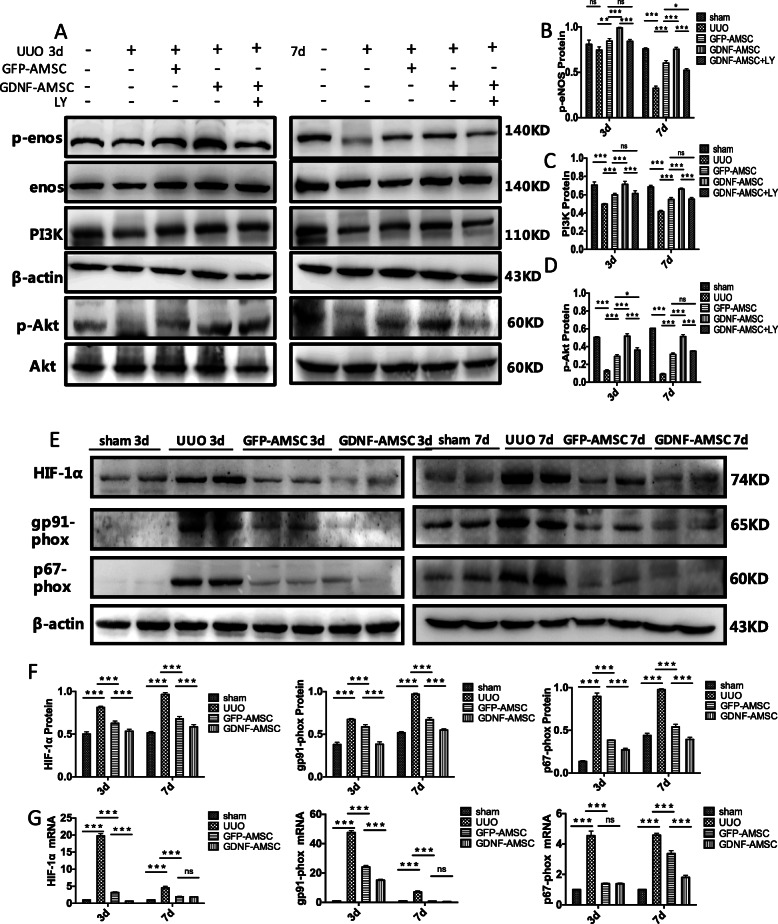
Western blot analysis of p-eNOS, eNOS, PI3K, p-Akt, Akt, HIF-1α, gp91-phox, and p67-phox and comparisons of HIF-1α, gp91-phox, and p67-phox mRNA expressions in each group by qRT-PCR. Representative Western blots (**a**) and quantitative data (**b**–**d**) are presented (*P < 0.05, **P < 0.01, ***P < 0.001). Blockade of PI3K/AKT/eNOS pathway abolished GDNF stimulation of Akt and eNOS phosphorylation. HIF-1α, gp91-phox, and p67-phox expression was confirmed after GDNF-AMSC treatment. Representative Western blot (**e**) and quantitative data for HIF-1α, gp91-phox, and p67-phox (**f**) are presented, and HIF-1α, gp91-phox, and p67-phox mRNA levels were shown by real-time PCR (**g**) (*P < 0.05, **P < 0.01, ***P < 0.001)

Since PI3K/Akt/eNOS pathway is a well-established mechanism for vascular remodeling and angiogenesis, additional experiments were conducted to determine these as possible molecular targets for GDNF-AMSCs. We found that the PI3K protein level was significantly decreased in the UUO group at each time point. As expected, phosphorylation of eNOS and Akt were also suppressed, but these inhibitions were reversed by GFP-AMSCs. Moreover, GDNF-AMSC-treated mice showed higher expressions of phosphorylated Akt and eNOS compared with the GFP-AMSC group, indicating that GDNF preconditioned AMSCs presented a better activation of PI3K/AKT/eNOS signaling pathway. To further explore the mechanism that GDNF enhances the angiogenic ability of GFP-AMSCs through PI3K/AKT/eNOS pathway, the PI3K inhibitor, LY294002 (LY), was utilized. The decreased expressions of p-eNOS, PI3K, and p-Akt in the GDNF-AMSCs +LY group were performed to confirm that the administration of LY can sufficiently block PI3K/AKT/eNOS pathway and the inhibitor can abolish the GDNF stimulation of Akt and eNOS phosphorylation (Fig. 7a–d). Collectively, the data indicate that GDNF-AMSCs played a protective role in endothelial cells by activating the PI3K/AKT/eNOS pathway.

### GDNF-AMSCs attenuate tissue hypoxia and oxidative effects

We evaluated whether the improvement of microvascular injury could attenuate renal hypoxia and thereafter suppress oxidative stress. Comparing with the sham group, the protein expression of hypoxia-inducible factor-1α (HIF-1α) was significantly increased after UUO owing to the rarefaction of the PTC (*P* < 0.001 vs. sham). However, there is a dramatic decrease expression of HIF-1α in the GFP-AMSC group (*P* < 0.001 vs. UUO) and a further decrease after GDNF-AMSC treatment at days 3 and 7 (*P* < 0.001vs. GFP-AMSC), providing evidence that GDNF can enhance the ability of AMSCs to reduce renal hypoxia (Fig. 7e, f). The gene expression of HIF-1α detected by RT-PCR revealed similar trends to those observed in the Western blot analysis (Fig. 7g).

To further explore the mechanism underlying the antifibrotic effect of GDNF-AMSCs, we evaluated whether the therapeutic effect of GDNF-AMSCs on renal fibrosis was regulated by inhibiting oxidative gp91-phox and p67-phox, oxidative markers, which are the major sources of ROS and play the important role in generating superoxide and subsequently contribute to cell damage. The protein expressions of gp91-phox and p67-phox were significantly higher at 3 and 7 days post-UUO (*P* < 0.001 vs. sham), suggesting the enhanced oxidative effects in association with accentuation of renal hypoxia. As evidence of better suppressing of oxidative stress in GDNF-AMSC-administered mice, the oxidative markers were found to be reduced in the GFP-AMSC group (*P* < 0.001 vs. UUO) and further reduced in the GDNF-AMSC group (*P* < 0.001 vs. GFP-AMSC) (Fig. 7e, f). The gene expression of gp91-phox and p67-phox detected by RT-PCR revealed similar trends to those observed in the Western blot analysis. However, there were no significant differences in gp91-phox gene expression at day 7 post-surgery and p67-phox gene expression at day 3 post-surgery between the GFP-AMSC and GDNF-AMSC groups (Fig. 7g). These results suggest the superior anti-oxidative effect of GDNF-modified AMSCs.

### AMSCs alleviate EndMT in UUO models

Oxidative stress is a redox state with overgeneration of ROS in cells which can induce severe damages to cells and tissues. Furthermore, under oxidative stress, endothelial cells lose their endothelial characteristics and acquired mesenchymal phenotypes. Immunostaining was performed to identify cells undergoing EndMT based on the coexpression of endothelial cells (CD31) and myofibroblast (a-SMA) markers. In our study, the abundant expression of CD31+ endothelial cells and resident a-SMA+ myofibroblasts was shown, whereas no double-labeled cells were shown in the sham-operated kidney. Obstructed kidney exhibited a decrease of CD31+ endothelial cells, and the coexpressing CD31+a-SMA+ cells were apparent, indicating an active role for EndMT progression in the fibrotic kidneys after UUO. However, GFP-AMSC therapy effectively inhibited EndMT by upregulating the expression of CD31 and decreasing the number of mesenchymal-like cells. Besides, the coexpressing cells within the renal interstitium were substantially reduced in the GDNF-AMSC group in comparison with the GFP-AMSC group (Fig. 3b). These results suggest that the EndMT process may be an important pathway contributing to renal fibrosis in UUO models, but GDNF-AMSC can alleviate EndMT progression.

## Discussion

Recently, the role of renal microvascular injury has been highlighted as one of the many causes of CKD [6, 25]. In our current study, we managed to find out that renal interstitial fibrosis has a close relation to the rarefaction of peritubular capillaries induced by the obstructive kidney. AMSCs’ treatment could alleviate the fibrotic process that was likely primarily attributed to increasing PTC density and relieving tissue hypoxia. Along with the improvement of renal microcirculation, excessive ROS production was reduced, which eventually was resulting in the inhibition of endothelial-myofibroblast transition. However, GDNF, as a tissue morphogen, showed not only a better effect on mitigating renal fibrosis in vivo but also better improvement of angiogenesis and reduction of endothelial damage caused by hypoxia in vitro.

Stem cell-based therapy is not only the most advanced regenerative therapy to date but one that has shown great promise for the treatment of CKD [8, 26]. There are different types of stem cells available for regenerative therapy. Despite their potent differentiation capacity, the wide use of embryonic stem cell (ESCs) and human induced pluripotent stem cells (hiPSCs) are both limited by the ethical issue and high tumorigenicity. AMSCs obtained from adipose tissue that possess the ability to differentiate along three main cell lineages are characterized by expressions of CD105, CD90, and CD73 and lack of expression for the hematopoietic markers CD34, CD45, and HLA-DR. More specifically, the quality and functions of MSCs obtained from adipose tissue of renal disease patients were not affected by exposure to uremic conditions, suggesting that it is safe and feasible to use autologous MSCs for CKD patients [27]. AMSCs could improve endogenous repair through direct differentiation into target cells or paracrine activity. To certify its differentiation capacity into endothelial cells, VEGF and FGF conditioned medium was used to induce AMSCs to differentiate towards an endothelial-like phenotype. The effective functions of exogenously administrated AMSCs have been reported in various models of acute and chronic kidney injury. A study demonstrated that adipose-derived mesenchymal stem cells (ADMSC) could be preserving kidney function after acute IR injury by reducing inflammation and oxidative stress [28]. Another study suggested that MSCs derived from adipose tissue (AD-MSCs), a less invasive and highly available source of MSCs, have an important therapeutic effect on renal function in 5/6 nephrectomy mice model [29]. In our study, GFP-AMSCs could successfully be located in the injured kidney through the blood circulation detected by a laser confocal microscope and maintain anti-fibrotic properties through promoting angiogenesis, which further suppresses tissue hypoxia, oxidative stress, and EndMT process.

The glial cell line-derived neurotrophic factor (GDNF), a survival-promoting molecule for midbrain dopaminergic neurons, was originally identified as a potential therapeutic agent for the treatment of neurodegenerative diseases. Afterward, several studies have reported further roles outside the nervous system [30, 31]. A study [32] found that kidney agenesis or dysgenesis in the GDNF −/−mutant mice may result from the promotion of GDNF on ureteric branching, suggesting that GDNF acts as a morphogen in kidney development. In the kidney, GDNF expression is associated with epithelial differentiation of the nephrogenic mesenchyme, and the absence of this gene results in renal aplasia or severe hypodysplasia [16, 33]. Therefore, this trophic factor raised great expectations of organogenesis. In our previous study, we found that the GDNF gene could enhance stem cell differentiation capacity and help stem cells better restrain cell apoptosis. Therefore, we explore if preconditioning AMSCs with the GDNF gene can enhance their differentiation, cell repairment, and angiogenic capacity and consequently maximize the anti-fibrotic effect of GFP-AMSCs transplantation.

Proper vascularization is essential for maintaining tissue well-being and functionality. Our previous research has reported that GDNF contributes to the progression of differentiation by confirming the ability of GDNF gene-engineered amniotic fluid-derived stem cells to differentiate into renal tubular epithelial-like cells [19]. In the current study, GFP-AMSCs exhibit cobbled-like rather than fibroblast-like morphology after being cultured in a conditioned medium for 21 days, and fluorescently labeled endothelial markers were observed in both GFP-AMSC and GDNF-AMSC groups, suggesting that AMSCs could differentiate into target endothelial cells. This can indicate that AMSCs have the potential to play the role of endothelial cells that create microvessels. Although the molecular mechanisms of angiogenesis are currently ambiguous, there is a piece of evidence strongly supporting the significant role of growth factors in this process, including VEGF, the most potent inducer of angiogenesis, and IGF-1, a contributor to endothelial function maintaining. A study of our group [34] showed more secretion of growth factors in the supernatant of GDNF-AMSC culture media, suggesting a potent potential of promoting angiogenesis, which was later verified by the Matrigel-based tube formation assay. In addition to that, endothelial cell-cell contacts are the guarantee for maintaining homeostasis of microvessels. Damage to endothelial cell-cell junctions contributes to increased microvascular permeability. Several leukocytes and reactive oxygen species (ROS) stimulated by hypoxia migrate through the injured endothelial cells into the renal interstitium, resulting in the swelling of the interstitial space and compression of the microvasculature to further aggravate hypoxia and eventually into the vicious cycle. VE-cadherin, representing endothelial adhering assembly and barrier architecture, was decreased in hypoxia condition. Nevertheless, the contacts within endothelial cells that cultured with the supernatant of GDNF-AMSC culture media could be better remodeled compared with that in the GFP-AMSC group. The beneficial effect of GDNF-AMSCs in improving microcirculation in vitro was able to be demonstrated through these data.

Several studies have detected capillary rarefaction in various models of progressive kidney disease, indicating that it is essential to maintain a normal tissue blood supply for the kidneys [35–37]. To further explore the relationship between microvascular injury and renal fibrosis, the novel experimental technique FMA was used to detect the PTC density, individual capillary cross-sectional area, and perimeter. FMA relies on low-melting-point agarose added with FluoSpheres to provide a visualization of microvasculature with a confocal laser microscope35. It should also enable us to quantify microvascular perfusion characteristics by using a MATLAB-based script, which could automatically generate an analysis of the microvasculature [24]. We found that the microvascular density and the single microvascular filling in the UUO models were significantly lower than those in the sham group, and accompanied by increased fibrosis marked by α-SMA, emphasizing a direct link between renal fibrogenesis and loss of peritubular perfusion. Therefore, we imagine that renal fibrosis can be alleviated by improving the renal microcirculation. According to experiments in vitro, we verify that AMSCs can repair endothelial cell injury and promote angiogenesis, whereas GDNF can enhance this effect. Therefore, we intravenously injected GDNF-modified AMSCs into the UUO models and observed that they can successfully be located in the kidney. Our study also showed that GDNF-AMSC treatment could better restore renal blood vessels, stimulate an augment in PTC density, and further relieve hypoxia compared with the GFP-AMSC treatment. Interestingly, the single cortical capillary surface area and perimeter in the GFP-AMSC group did not differ significantly compared with the UUO group, whereas they showed a more dramatic increase after GDNF-AMSC treatment, suggesting that some capillaries might lack perfusion in the GFP-AMSC group. Thus, an advantage of GDNF-AMSCs in alleviating renal fibrosis may be due to its ability to increase peritubular microvasculature.

Although many reasons are inducing CKD, renal hypoxia secondary to microvascular injury represents a major cause contributing to tissue damage and loss of renal function [38, 39]. The kidney is rich in vascular structure and is a highly vascularized viscera. Therefore, it is essential to maintain a normal tissue blood supply for the kidneys. As a common cause of CKD, capillary rarefaction results in the imbalance of local tissue oxygen supply and demand together with accumulation of waste products of metabolism, which further lead to a series of events, such as epithelial cell injury and the accumulation of reactive oxygen metabolites, as well as inflammation and oxidative stress [18, 35, 40]. Oxidative stress contributes to lipid peroxidation, increased hydrogen peroxide, and DNA and protein damage. Furthermore, excessive ROS production can aggravate the damage of endothelial cells and further lead to endothelial-to-mesenchymal transition (EndMT). Several studies have observed endothelial cells that acquired the structural and functional characteristics of mesenchymal cells after kidney injury demonstrating myofibroblasts may originate from endothelial cells, so-called EndMT [41, 42]. In our study, endothelial cells undergoing EndMT in the UUO models were observed, demonstrating that injured endothelial cells can transform into myofibroblasts. In contrast, this EndMT process was suppressed after GFP-AMSCs and GDNF-AMSC treatment by noting that fewer CD31+ endothelial cells and a-SMA+ myofibroblasts colocalized in the renal interstitium. These data indicate that GDNF-AMSCs can inhibit the progress of EndMT caused by oxidative stress.

The role of GDNF-AMSC treatment in renal fibrosis is demonstrated in vitro and in vivo, which is a significant step before the clinical translation of the GDNF-AMSCs. However, we should acknowledge some limitations of our study. First, stem cells cultured to the late-stage exhibit some signs of aging, phenotypic changes, and tumorigenesis property. Additionally, genetic modification of AMSC may have a safety issue, which may cause unpredictable clinical consequences. Therefore, future studies are needed to prove the safety and effectiveness of GDNF-AMSCs before their clinical application.

Several studied have reported that PI3K/Akt signaling pathway plays a critical role in cell growth, differentiation, proliferation, and apoptosis [43–45]. PI3K, as one of the key signal transducer implicated in angiogenesis, delivers signals from receptor tyrosine kinases to downstream factor, Akt. In the current study, GDNF-AMSC increased the expression of proangiogenic cytokine and VEGF. These increased our interest in whether GDNF-AMSCs protect renal tissues against microvascular injuries via activation of the PI3K/Akt signaling pathway. To explore the mechanism underlying the angiogenic effect of GDNF-AMSCs, western blot analysis of PI3K and phosphorylated Akt was detected to confirm that GDNF-AMSC could better activate PI3K/Akt pathways compared to fibrotic kidney treated with GFP-AMSCs. Further, GDNF-AMSCs upregulated the phosphorylation of Akt and specific inhibition of PI3K abolished the attenuating effect of GDNF-AMSCs on the impaired endothelial nitric oxide (NO) synthase (eNOS). ENOS is an important downstream target of the PI3K/Akt pathway. Activated Akt phosphorylates eNOS, leading to the improvement of endothelial function. These results revealed PI3K/Akt/eNOS cascades as fundamental signaling mediating the angiogenesis effect of GDNF-AMSCs.

## Conclusion

In summary, we intend to show that the GDNF gene enhances the ability of AMSCs in improving renal microcirculation through the PI3K/Akt/eNOS signaling pathway. As a result, the GDNF-modified AMSCs can further alleviate renal hypoxia and oxidative stress secondary to microvascular injury and inhibit the EndMT process and kidney fibrogenesis, which should have wide implications in designing future remedies for the treatment of CKD. These studies represent the attempt to molecularly characterize the relationship between microvascular injury and renal fibrosis and successfully applied the novel quantitative imaging tool to kidney diseases.

## Supplementary Information

**Additional file 1.**

## Data Availability

The datasets generated/analyzed during the current study are available.

## Abbreviations

AMSCs: Adipose mesenchymal stem cells
ECM: Extracellular matrix
FMA: Fluorescence microangiography
GDNF: Glial cell line-derived neurotrophic factor
GFP: Green fluorescence
HIF-1a: Hypoxia-inducible factor-1 a
UUO: Unilateral ureteral occlusion
VEGF: Vascular endothelial growth factor
α-SMA: α-Smooth muscle actin
CKD: Chronic kidney disease
ESRD: End-stage renal disease
PI3K: Phosphoinositide 3 kinase
PTC: Peritubular capillary
