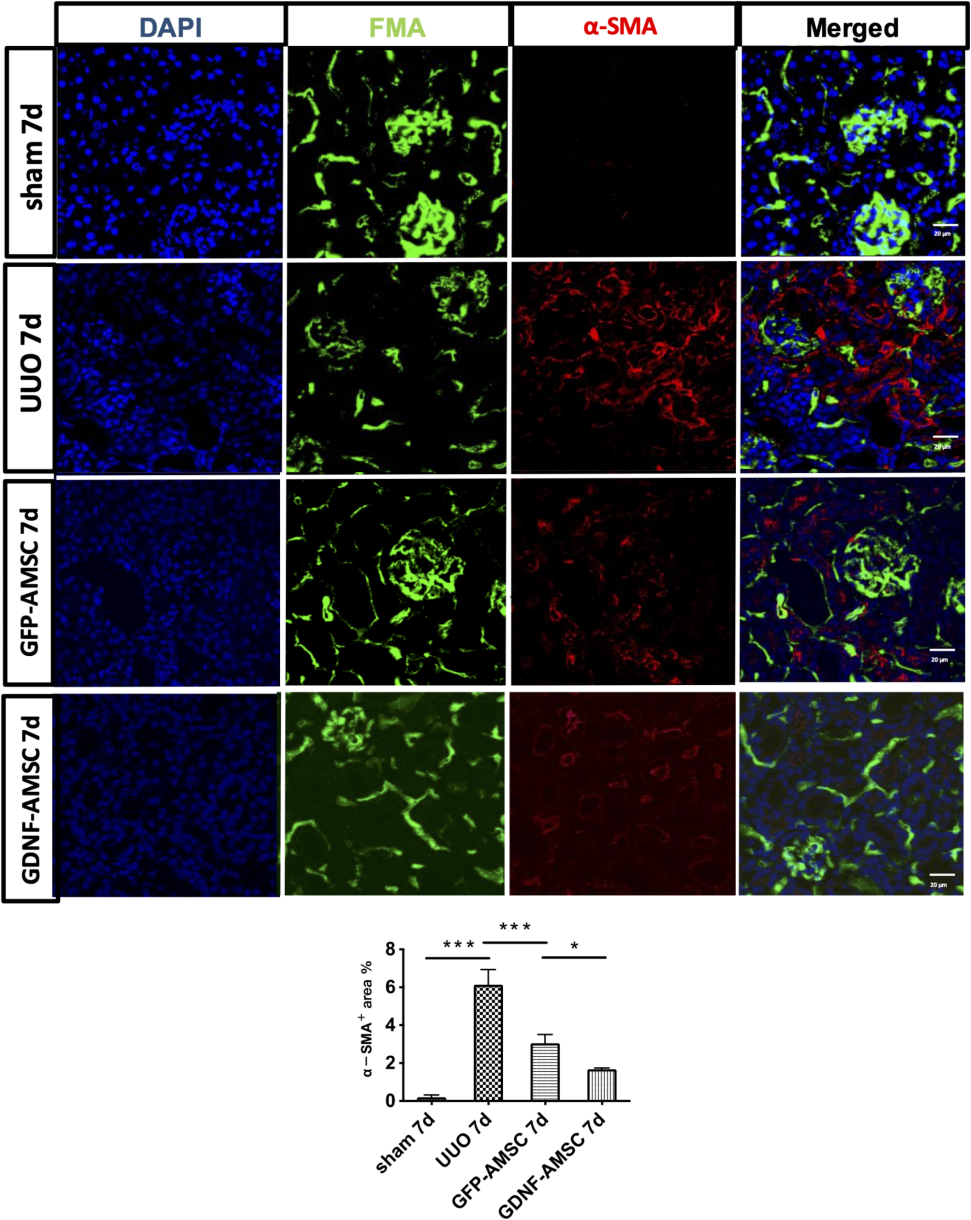# Correction: Enhanced renoprotective effect of GDNF-modified adipose-derived mesenchymal stem cells on renal interstitial fibrosis

**DOI:** 10.1186/s13287-025-04335-0

**Published:** 2025-04-20

**Authors:** Shulin Li, Yanping Wang, Zhuojun Wang, Lu Chen, Bangjie Zuo, Caixia Liu, Dong Sun

**Affiliations:** 1 Department of Nephrology, Affiliated Hospital of Xuzhou Medical University, 99 West Huai-hai Road, Xuzhou, 221002 Jiangsu China; 2 Department of Internal Medicine and Diagnostics, Xuzhou Medical University, Xuzhou, 221002 China

**Stem Cell Research & Therapy (2021) 12:27**

10.1186/s13287-020-02049-z

Upon a recent review of the published version and comparison with the original data, the authors have identified two image-related errors that require correction:

1. Figure 1A (AMSCs image): The image representing AMSC morphology was inadvertently misused during figure preparation. The authors have now prepared the correct image to replace the erroneous one.

**Figure:**
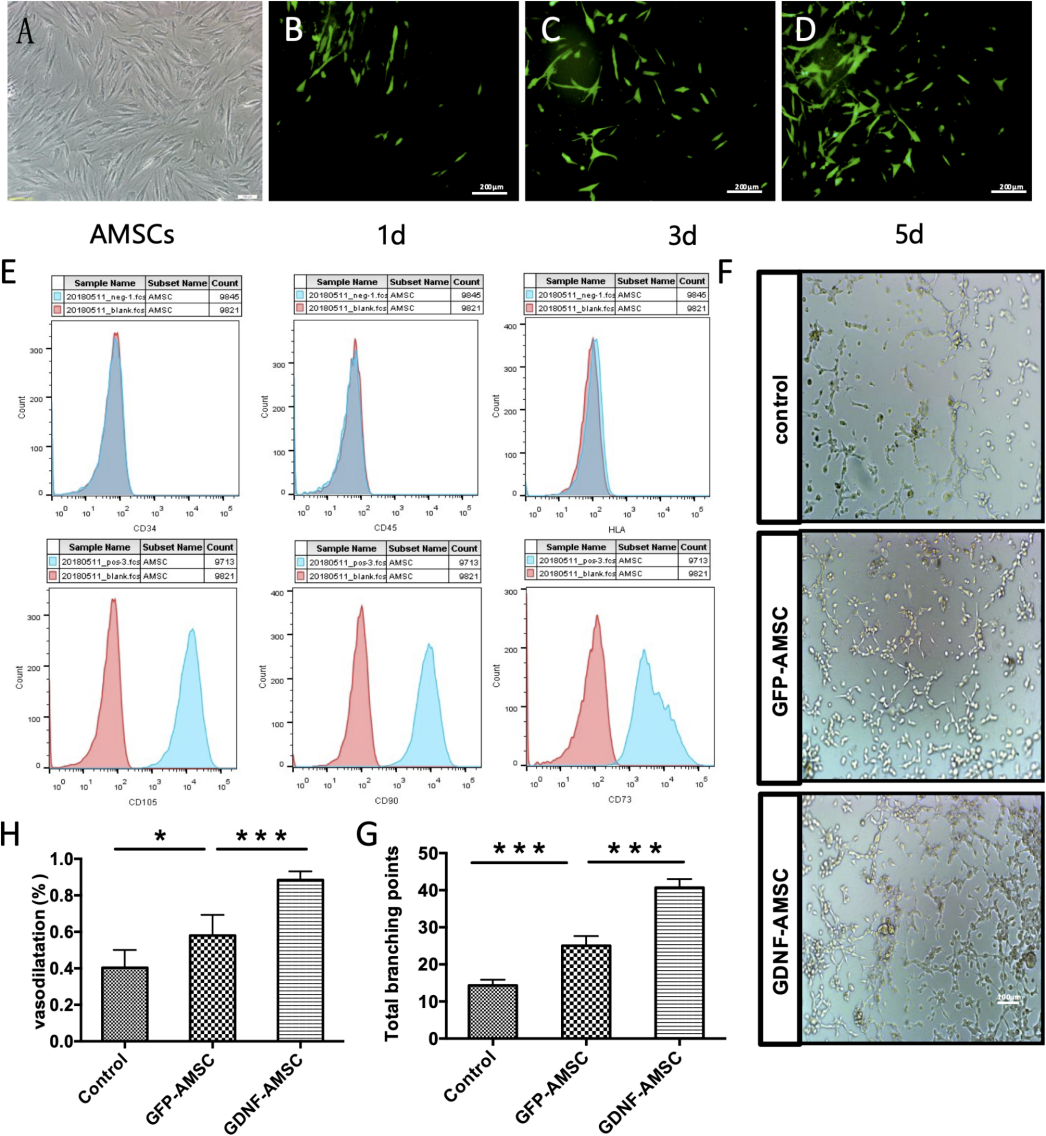

2. Figure 6 (GDNF-AMSCs group): The image for the GDNF-AMSCs group was mistakenly included. After reviewing the original data, the authors have identified the accurate image and prepared a corrected version. In addition, the corresponding statistical graph in Figure 6 has also been updated to reflect the corrected data. Both the corrected image and the revised statistical chart are included in the revised figure.

**Figure:**