# Supplementary material for: Enhanced renoprotective effect of GDNF-modified adipose-derived mesenchymal stem cells on renal interstitial fibrosis
Source: Stem Cell Res Ther. 2021 Jan 7;12:27. doi: 10.1186/s13287-020-02049-z (PMC7792009; doi:10.1186/s13287-020-02049-z)
Supplement: Supplementary file 1 — Additional file 1. [file 13287_2020_2049_MOESM1_ESM.pdf]

Supplementary figure S1

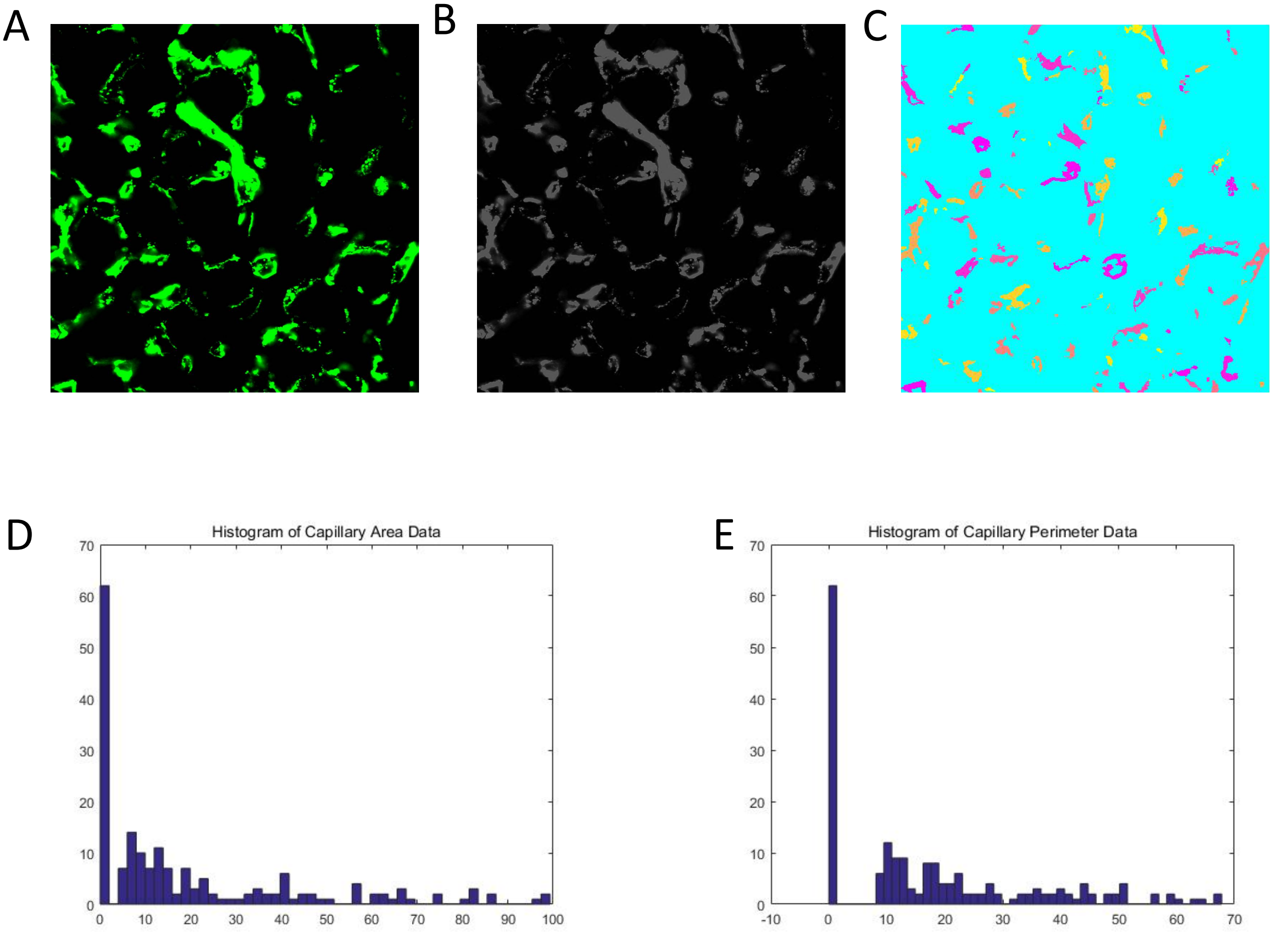

Supplementary figure S1: Capillaries were analysed by MATLAB script
Openning each green channel picture in Image J and splitting them into the grayscale (B). this picture will be analyzed by the MATLAB script which automatically generates 4 images, including the original image (A), the binary image with different colors (C), the histograms of the area data and perimeter data (D and E). The MATLAB script also automatically generates two excel sheets with number, area (μm^2) and perimeter (μm) of the capillaries for further analysis by Graphpad software. The MATLAB script was set at a require value (cut-off values $<4.9\mu m^2$ and $>100\mu m^2$ in our case) in advance. So the capillaries that do not meet the requirements will be measured as zero.
